# Statistics of Cholera

**Published:** 1849-01

**Authors:** 


*Statistics of Cholera.*—For the following statistics of cholera, in other countries, quoted from various periodicals, we are chiefly indebted to the *Medical News and Library.* It will be perceived that the ratio of mortality, in different countries, is remarkably uniform, being a little over one half the number of cases. The bearing of this fact upon the subject of treatment is deserving of notice.

"In *Russia,* between the 28th of October, 1846, the period of the commencement of the epidemic, and the 5th of July, 1848, 290,318 persons were attacked, of whom 116,668 died."

*Berlin.*—"From the 27th of July, to the 11th of October, 1848, the numbers have been as follows:—Numbers of cases, 2009 Deaths, 1262. Recoveries, 472. Under treatment, 275."

*Hamburgh.*—"The official report states that up to the 9th inst., the total number of persons attacked, was 2,229, of whom 1,043 had, up to that day, fallen victims; that 411 remained under treatment, and 775 had been cured.'

*Turkey.*—"The accounts received from *Constantinople* by the last mail, announced that the cholera is gaining 'ground, the daily mortality averaging nearly 200."

At *Smyrna*, total attacked, 3,212. Total of deaths, 2,494.

*Syria and Palestine.*—"At *Damascus*, the deaths are reported as varying from 500 to 600 daily. *Aleppo* averages 150. *Antioch* about 50 daily."

*The Hague.*—"In the cholera hospital 44 patients have been received; of these, 18 have died, 3 have recovered, and the remainder are under treatment."

*Scotland.*—"Up to Nov. 7, 468 cases had been reported; deaths, 243; recoveries, 54."

*Cholera at New York.*—The disease made its appearance early in December, at the Quarantine ground, Staten Island, among the passengers by the ship New York, which had just arrived from Havre. It broke out among, and has been for the most part restricted, to the German passengers by the New York A considerable proportion of the cases that have occurred at Staten Island, have occurred among patients in the marine hospital, who were inmates of the hospital before the arrival of the ship New York, and who had no intercourse with the cholera patients from that vessel. One case only, has, as yet, occurred in the city, the patient having had no intercourse with the Quarantine. We hope full and accurate statistics will be preserved and reported, not only of the number of cases and deaths, but of those who were in intercourse, direct or indirect, with patients laboring under the disease, both before going on board the vessel New York, and at the Quarantine. Statistics on the latter point are highly important as regards the question of contagion. We have not taken pains to collect all the daily reports of the Quarantine Physician, Dr. Whiting, to the Mayor of New York; but we have before us these reports from December 7th to 20th, inclusive. During that period 44 cases occurred, and 20 deaths. Of these cases, it is stated, that 24 were passengers by the vessel New York, and 7 had no connexion with the passengers by that vessel. As regards the remaining 13, the reports are not explicit. The disease is still progressive.

The necessity of Quarantine regulations, in order to prevent the diffusion of the disease, is an important question. Its importance to those in Quarantine, as well as to those who are apprehensive of infection, should not be overlooked. It seems really a needless hardship, to detain all the passengers of the ill-fated New York, until the major part die with the disease. Are not the circumstances connected with their detention in Quarantine, calculated to predispose to the disease? Since probably not one in ten of Physicians, and the public, regard the disease as contagious, it strikes us as more than an absurdity to act in direct opposition to this opinion, when the inhumanity of such a course is but too obvious.

*Cholera at New Orleans.*—It seems now (28th Dec.) pretty certain that the disease has made its appearance at New Orleans. On the 23d, seventy-nine had been reported since the 20th, at the Charity Hospital, and new cases were hourly occurring. Several merchants of the city had died with the disease. The citizens, with the selfishness so apt to be developed by the apprehensions of a pestilential disease, are clamorous for Quarantine restrictions, and endeavoring to flee from the disease. There is no evidence, in the reports thus far received, that the disease was imported from abroad.

